# FHLdb: A Comprehensive Database on the Molecular Basis of Familial Hemophagocytic Lymphohistiocytosis

**DOI:** 10.3389/fimmu.2020.00107

**Published:** 2020-01-31

**Authors:** Laura Viñas-Giménez, Natàlia Padilla, Laura Batlle-Masó, Ferran Casals, Jacques G. Rivière, Mónica Martínez-Gallo, Xavier de la Cruz, Roger Colobran

**Affiliations:** ^1^Immunology Division, Hospital Universitari Vall d'Hebron (HUVH), Vall d'Hebron Research Institute (VHIR), Department of Cell Biology, Physiology and Immunology, Autonomous University of Barcelona (UAB), Barcelona, Spain; ^2^Jeffrey Model Foundation Excellence Center, Barcelona, Spain; ^3^Research Unit in Clinical and Translational Bioinformatics, Vall d'Hebron Research Institute (VHIR), Barcelona, Spain; ^4^Servei de Genòmica, Departament de Ciències Experimentals i de la Salut, Universitat Pompeu Fabra, Parc de Recerca Biomèdica de Barcelona, Barcelona, Spain; ^5^Departament de Ciències Experimentals i de la Salut, Institute of Evolutionary Biology (UPF-CSIC), Universitat Pompeu Fabra, Parc de Recerca Biomèdica de Barcelona, Barcelona, Spain; ^6^Pediatric Infectious Diseases and Immunodeficiencies Unit (UPIIP), Hospital Universitari Vall d'Hebron (HUVH), Vall d'Hebron Research Institute (VHIR), Universitat Autònoma de Barcelona (UAB), Barcelona, Spain; ^7^Institut Catala per la Recerca i Estudis Avançats (ICREA), Barcelona, Spain; ^8^Genetics Department, Hospital Universitari Vall d'Hebron (HUVH), Barcelona, Spain

**Keywords:** primary immunodeficiency, hemophagocytic lymphohistiocytosis, database, genetics, mutation, genetic variant

## Abstract

**Background:** Primary immunodeficiencies (PIDs) are a heterogeneous group of disorders. The lack of comprehensive disease-specific mutation databases may hinder or delay classification of the genetic variants found in samples from these patients. This is especially true for familial hemophagocytic lymphohistiocytosis (FHL), a life-threatening PID classically considered an autosomal recessive condition, but with increasingly demonstrated genetic heterogeneity.

**Objective:** The aim of this study was to build an open-access repository to collect detailed information on the known genetic variants reported in FHL.

**Methods:** We manually reviewed more than 120 articles to identify all reported variants related to FHL. We retrieved relevant information about the allelic status, the number of patients with the same variant, and whether functional assays were done. We stored all the data retrieved in a PostgreSQL database and then built a website on top of it, using the Django framework.

**Results:** The database designed (FHLdb) (https://www.biotoclin.org/FHLdb) contains comprehensive information on reported variants in the 4 genes related to FHL (*PRF1, UNC13D, STXBP2, STX11*). It comprises 240 missense, 69 frameshift, 51 nonsense, 51 splicing, 10 in-frame indel, 7 deep intronic, and 5 large rearrangement variants together with their allelic status, carrier(s) information, and functional evidence. All genetic variants have been classified as pathogenic, likely pathogenic, uncertain significance, likely benign or benign, according to the American College of Medical Genetics guidelines. Additionally, it integrates information from other relevant databases: clinical evidence from ClinVar and UniProt, population allele frequency from ExAC and gnomAD, and pathogenicity predictions from well-recognized tools (e.g., PolyPhen-2, SIFT). Finally, a diagram depicts the location of the variant relative to the gene exon and protein domain structures.

**Conclusion:** FHLdb includes a broad range of data on the reported genetic variants in familial HLH genes. It is a free-access and easy-to-use resource that will facilitate the interpretation of molecular results of FHL patients, and it illustrates the potential value of disease-specific databases for other PIDs.

## Introduction

Primary immunodeficiencies (PIDs) are a heterogeneous group of disorders affecting the immune system. Most PIDs are considered to have a monogenic cause, but incomplete penetrance, variable expressivity, and interactions between genetic and environmental factors can contribute to their phenotypic diversity. The diagnostic workup for PIDs is based on the patients' clinical manifestations and the results of complex laboratory techniques to guide selection of the candidate gene or genes to be tested (1). However, because of the diverse nature of these conditions, candidate gene selection is not usually straightforward. Hence, the development of massive parallel sequencing or next-generation sequencing (NGS) is rapidly replacing direct sequencing (Sanger method) as the first-choice method for genetic diagnosis of PIDs (2). Several NGS-based approaches, from whole-genome sequencing to specific PID panels, are currently in use, and they have led to major breakthroughs in the diagnosis of these disorders (3–6).

Nonetheless, the huge amount of data obtained by NGS technologies makes analysis and interpretation of the results a cumbersome process compared with conventional genetic testing. The clinical significance of a potentially relevant variant may be difficult to ascertain if there is no easily accessible information to consult. Review of the literature is an option, but it can be time-consuming and sometimes, exhausting. The lack of uniform criteria across studies to establish the pathogenicity of genetic variants further complicates this task.

Disease-specific databases, mainly known as locus-specific databases (LSDBs), may be the best tool to help professionals involved in genetic analysis. LSDBs must be comprehensive in collecting genetic variants relevant for a disease and must be rigorous in providing evidence that supports the role of the variants. Ideally, LSDBs should interact with other established databases and computational tools to depict an overall view of each variant. Unfortunately, LSDBs for PIDs are scarce, often outdated, and not user-friendly. This study was developed when we realized that there was no LSDB for familial hemophagocytic lymphohistiocytosis (FHL), an interesting and complex PID, whose genetic basis is currently under discussion (evolving from classical autosomal recessive inheritance to other models).

Hemophagocytic lymphohistiocytosis (HLH) comprises a group of rare disorders characterized by a highly stimulated but ineffective immune response that typically produces a massive proinflammatory cytokine storm resulting in fever, pancytopenia, hemophagocytosis, central nervous system (CNS) dysfunction, and multiorgan failure (7). It most frequently affects infants at early ages, but the disease is also observed in children and adults of all ages. If left untreated, the disease may rapidly progress and lead to death in a few weeks. Treatment for this condition includes immunosuppressive and immune-modulating therapy to control the inflammation and organ damage, but in the most severe cases, hematopoietic stem cell transplantation (HSCT) is the definite cure (8).

HLH presents in a wide spectrum of clinical contexts, including fever of unknown origin, acute liver failure, sepsis-like, Kawasaki-like, and neurologic abnormalities (8). Although a distinctive constellation of clinical and laboratory features has been described for HLH, diagnosis remains challenging as patients may have very different clinical manifestations associated with a variety of triggers (9). Atypical presentations involving mainly CNS or chronic pathologic inflammation manifestations are examples of such complexity (10–12).

HLH patients are often categorized as having either primary/familial or secondary/sporadic HLH. Familial HLH (FHL) is caused by biallelic mutations in genes involved in the granule-dependent exocytosis pathway, which lead to impaired natural killer (NK) and T-cell cytotoxic activity. To date, 4 genes (*PRF1, UNC13D, STXBP2*, and *STX11*) and 1 genomic region (9q21.3–22) have been identified as candidate causes of FHL2 (13), FHL3 (14), FHL4 (15), FHL5 (16), and FHL1 (17), respectively (18). Other monogenic diseases that produce HLH are Chédiak-Higashi syndrome (*LYST*), Griscelli syndrome type 2 (*RAB27A*), Hermansky-Pudlak syndrome (*AP3B1*), X-linked lymphoproliferative syndrome (XLP)-1 (*SH2D1A*), and XLP-2 (*XIAP*) (19). HLH can also develop in the absence of familial recurrence and without biallelic mutations in the causal genes, usually in the context of infections, malignancies, and autoinflammatory or metabolic diseases. These are considered “secondary” or “sporadic” HLH (sHLH) (20). In recent years and coinciding with the rapid evolution of NGS technology, the number of studies reporting new HLH-related variants has significantly increased (21, 22), and these have elucidated that a proportion of sHLH patients with or without a functional defect harbor monoallelic variants in one of the FHL-related genes (23, 24). In addition, novel types of inheritance have been proposed for HLH, including polygenic and dominant transmission models (25–28).

A percentage of the reported HLH variants can be found in generalist databases such as ClinVar, OMIM (Online Mendelian Inheritance in Man), LOVD (Leiden Open Variation Database), and HGMD (Human Gene Mutation Database). However, these databases may be incomplete, outdated, or not manually curated. As was mentioned, there is no dedicated LSDB for FHL genes, and this may lead to delays in classifying the genetic variants found in FHL patients and, consequently, in their diagnosis and treatment.

Here, we present FHLdb, a comprehensive database on the molecular basis of familial HLH. FHLdb is a web-based open-access repository of reported variants in the *PRF1, UNC13D, STXBP2*, and *STX11* genes. FHLdb provides detailed information on each variant, including functional evidence of pathogenicity when available, and enables links with other widely used databases (e.g., ClinVar, dbSNP, ExAC, gnomAD) to provide users with the most easily accessible and complete genetic information related to this condition.

## Methods

### Bibliographic Data

A systematic search in the medical literature retrieved more than 120 related articles, which were manually reviewed to collect all reported variants in FHL patients. Literature search was mainly performed using the Medline database from the National Library of Medicine through the PubMed search engine. PubMed is a free resource supporting the search and retrieval of peer-reviewed scientific literature. Combinations of the following keywords have been used for the search procedure: HLH, FHL, hemophagocytic lymphohistiocytosis, familial hemophagocytic lymphohistiocytosis, hemophagocytic syndrome, PRF1, UNC13D, STXBP2, STX11, mutation, variant, genetic variant, biallelic, monoallelic, heterozygous, homozygous, etc.

From these studies, we also collected other relevant information to include in FHLdb, such as the reported status of the variant (biallelic and/or monoallelic), whether the variant was found in more than one patient, and whether a functional assay had been carried out to determine the consequences of the variant.

### Database Set-Up

At the technical level, to guarantee user-friendly access to the data, we stored all information in a PostgreSQL database and then built a website on top of that, using the Django framework. Finally, we completed the information on each variant by providing links to other databases of interest.

## Results

**Figure 1:**
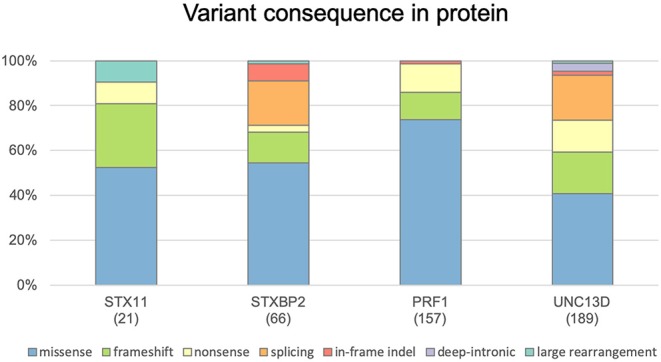
Distribution of genetic variants according to their effect at the protein level. Histograms show the distribution of *STX11, STXBP2, PRF1*, and *UNC13D* variants included in FHLdb. The total number of variants reported in each gene is indicated within parentheses.

**Figure 2:**
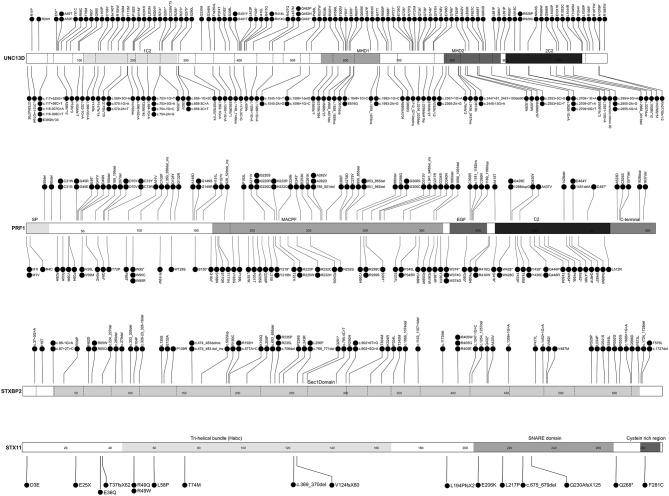
FHLdb genetic variants in the *UNC13D, PRF1, STXBP2*, and *STX11* genes. Linear representation of UNC13D, PRF1, STXBP2, and STX11 at the protein level. The diagram shows the distribution and location of all variants reported in FHLdb, except for large genomic rearrangements. Dotted lines delimitate each exon. Functional domains are indicated above each gray-scale colored square. MHD1 and MHD2, Munc13 homology domains 1 and 2; SP, signal peptide; MACPF, perforin membrane attack complex; EGF, epidermal growth factor-like; C2, calcium binding domain. The figure was designed using the Protein Paint free software included in the St. Jude PeCan Data Portal (https://pecan.stjude.cloud) (29).

FHLdb (https://www.biotoclin.org/FHLdb) is a comprehensive collection of reported variants in the 4 genes known to cause FHL: *PRF1, UNC13D, STXBP2*, and *STX11*. The literature search yielded 433 different variants in these genes, distributed as follows: 240 missense, 69 frameshift, 51 nonsense, 51 splicing, 10 in-frame indel, 7 deep intronic, and 5 large rearrangements (Figure 1). *UNC13D* showed the largest number of variants (189), followed by *PRF1* (157), *STXBP2* (66), and *STX11* (21) (Figure 2).

Each variant was manually curated, and we followed the Human Genome Variation Society (HGVS) guidelines for variant nomenclature (http://varnomen.hgvs.org) to obtain a harmonized, interchangeable, clear dataset. We found that the nomenclature of 29% of the variants was incomplete or did not follow these guidelines in their original description; hence, these variants were renamed (Supplementary Table 1). In this manuscript, both “mutation” and “variant” terms have been used. While both terms indicate a change in the nucleotide sequence, in medicine “mutation” is used to indicate a sequence variant associated with a disease phenotype. On the other hand, the current guidelines of authoritative organizations recommend using neutral terms like “variant” followed by a classification term like pathogenic, benign, etc. (see HGVS website for extended discussion about terminology). Therefore, we preferably used “variant” and restricted the use of “mutation” in some specific cases to indicate disease-causing variants.

**Figure 3:**
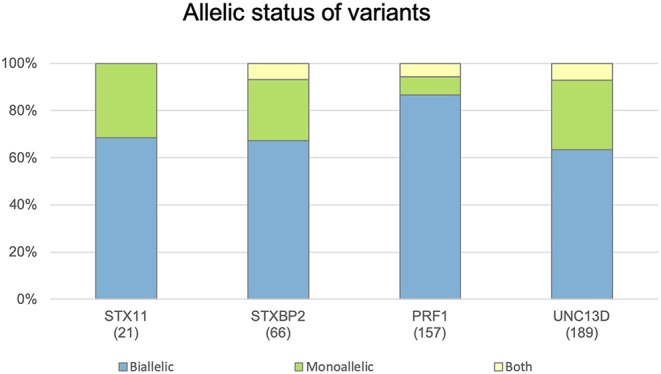
Distribution of genetic variants according their allelic status. Histograms show the distribution of variants in the *STX11, STXBP2, PRF1*, and *UNC13D* genes included in FHLdb. The total number of variants reported in each gene is indicated within parentheses.

The allelic status of each variant is reported in the following terms: biallelic, when it was reported in homozygous or compound heterozygous state, and monoallelic. Thus, a variant can be reported as biallelic, monoallelic, or both. Although most variants are found in biallelic state, because of the recessive inheritance of these 4 genes, a significant percentage (ranging from 12 to 36%) were reported as monoallelic (Figure 3), which indicates the increasingly recognized role of monoallelic variants in FHL (22, 23, 25, 28). However, caution must be exercised in evaluating the role of monoallelic variants: FHL is essentially an autosomal recessive disease and there is only one well-described example of monoallelic variants as disease causing mutations through a dominant-negative mechanism (28). The other reported monoallelic variants in FHL are not proven to be disease causing by itself but may represent susceptibility/risk factors in genetic predisposition to FHL (23, 30).

We also reviewed whether functional assays had been carried out to decipher the functional consequences of the variant. This is one of the main challenges in the interpretation of genetic variants, especially those that are not clearly related to loss of function (e.g., missense variants). Variants with reported functional studies are indicated and the corresponding reference is provided.

To help the interpretation, we manually classified all genetic variants according to the American College of Medical Genetics (ACMG) guidelines. ACMG recommends a five-tier system of classification using a specific standard terminology: pathogenic, likely pathogenic, uncertain significance, likely benign and benign (31).

FHLdb integrates information from other widely used databases, such as clinical evidence from ClinVar and UniProt, and population allele frequency from ExAC and gnomAD, and it provides computational pathogenicity predictions from PolyPhen-2, CADD, PON-P2, and SIFT. We found that 91% of the missense variants were predicted to be pathogenic by at least one *in silico* predictor, and 96% of the variants had an allele frequency below 1% in the ExAC database. Remarkably, only 20% of variants were included in the ClinVar database, the largest resource to support clinical variant interpretation. This finding indicates that there is still little available information in ClinVar about FHL genetic variants, and it underscores the need for disease-specific databases.

**Figure 4:**
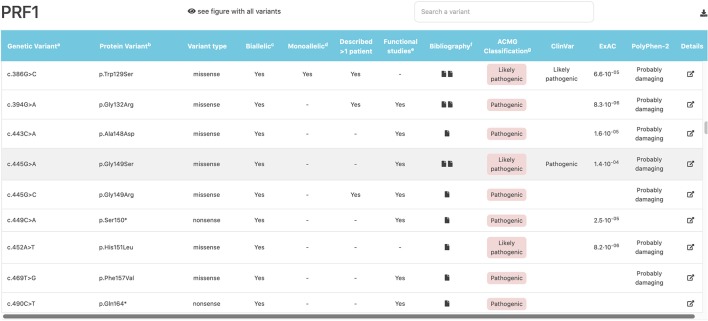
Example of the gene view page where all variants are listed and their main characteristics described. The *PRF1* gene view page is shown. The variant indicated by the pointer is highlighted in gray (in this example, c.445G>A).

**Figure 5:**
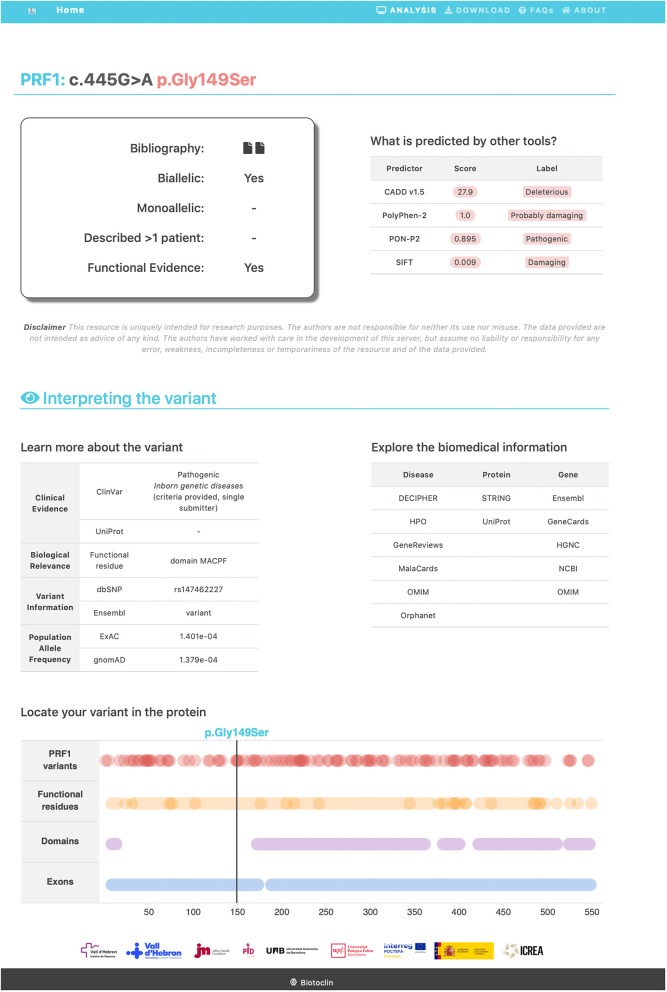
Example of the detailed information page, containing specific information about a variant. A missense variant in the *PRF1* gene is shown.

All the information obtained was compiled in an open, user-friendly website (https://www.biotoclin.org/FHLdb), divided into three main parts. First, there is a welcome page presenting the database, where the user finds the 4 genes typically associated with FHL (*PRF1, UNC13D, STXBP2, STX11*), a description of the information available for each variant, and other general information. Second, after selecting the gene of interest, the user is transferred to the gene view page where all variants are listed and the main characteristics are shown (Figure 4). Third, a link to more detailed information located in the last column allows the user to access a section about the specific variant, which includes the following: (1) a summary of the references (at least the first) describing the variant, whether the variant has been reported as biallelic and/or monoallelic, whether it was found in more than 1 patient, and whether a functional assay was carried out; (2) the *in silico* pathogenicity predictions (CADD, PolyPhen-2, PON-P2, and SIFT); (3) links to other databases of interest regarding the specific variant (ClinVar, UniProt, dbSNP, Ensembl, ExAC, Gnomad) and databases containing general knowledge about the disease, the gene, and the protein (e.g., Decipher, GeneReviews, OMIM, GeneCards, NCBI); and (4) a diagram showing the location of the variant in the protein (Figure 5).

## Concluding Remarks

To our knowledge, this is the first LSDB for FHL syndrome. A prototypical definition of LSDB is “a collection of sequence variants in a specific gene(s) that causes a Mendelian disorder or change in phenotype” (32). FHLdb is intended to collect all known variants in the 4 genes typically associated with familial HLH published in peer-reviewed literature. Beyond simply listing all reported variants in the *PRF1, UNC13D, STXBP2*, and *STX11* genes, FHLdb includes other relevant data, such as the variant frequency in the general population, computational prediction of pathogenicity, and functional evidence. Additionally, it provided links to external reference databases to enable users to easily access all available related resources.

To build FHLdb, we followed general consensus recommendations to enable broad and easy to manage use (32, 33). FHLdb is an open-access resource, licensed under the Creative Commons Attribution-ShareAlike (4.0) International License, which allows users to adapt, remix, transform, and build upon the material for any purpose, even commercial intents, under the terms specified in the full-text license (https://creativecommons.org/licenses/by-sa/4.0/legalcode). All existing data in FHLdb can be downloaded in a user-friendly format (txt) and in other programming languages (e.g., Python, Curl, or Wget).

Curation is one of the greatest strengths of LSDBs and is likely the aspect that most differentiates them from general databases (34). Each variant in FHLdb was manually curated and almost one third of entries were renamed to adapt the nomenclature to the HGVS. We decided to include only genetic variants supported by peer-reviewed, indexed articles. Therefore, each variant has at least one associated article where the user can find further information beyond that included in FHLdb.

It is important that LSDB creators accept a long commitment and continued updating of the database. In our case, FHL/HLH is a strategic research field in our laboratory. We are currently working on two research projects focused on HLH, funded by Instituto de Salud Carlos III in Spain (see funding section), and we are determined to keep FHLdb updated in the future. To accomplish that, we will be alert to new publications on the genetic basis of HLH and we encourage users to report on a new variant by sending us the article describing it.

In this first version of FHLdb, we focused on genes involved in the cytotoxic pathway. Other genes causing congenital immunodeficiency syndromes that are associated with HLH (e.g., *NLRC4, LYST, RAB27A, SH2D1A, XIAP*) have not as yet been reported but it is planned to include them in FHLdb in the near future, expanding the data base to a compilation of genetic variants associated to HLH. The increasing use of NGS is rapidly expanding the number of genetic variants found in HLH patients. Implementation of FHLdb aims to help scientists and clinicians working in the field in their search for FHL-related genetic variants to support their research and daily clinical practice.

## Data Availability Statement

Publicly available datasets were analyzed in this study. All data is available in http://www.biotoclin.org/FHLdb/.

## Author Contributions

LV-G performed the literature search, selection, and manual curation of the genetic variant data, and wrote part of the manuscript. NP adjusted the variants to the HGVS guidelines, performed the technical database set-up and web development, and wrote part of the manuscript. LB-M, FC, JR, MM-G, and XC collaborated in the database design and literature search. RC was responsible for designing the study, supervising the literature search and database set-up, writing the manuscript, and approving the final draft. All authors reviewed the manuscript and contributed to the final draft.

### Conflict of Interest

The authors declare that the research was conducted in the absence of any commercial or financial relationships that could be construed as a potential conflict of interest.

## Supplementary Material

Supplementary Table 1 Variants renamed in FHLdb according the HGVS guidelines.
